# Endothelin-1 Induces Mesothelial Mesenchymal Transition and Correlates with Pleural Fibrosis in Tuberculous Pleural Effusions

**DOI:** 10.3390/jcm8040426

**Published:** 2019-03-28

**Authors:** Zhung-Han Wu, Jie-Heng Tsai, Cheng-Ying Hsieh, Wei-Lin Chen, Chi-Li Chung

**Affiliations:** 1 Division of Pulmonary Medicine, Department of Internal Medicine, Taipei Medical University Hospital, Taipei 110, Taiwan; eddiemanstein@yahoo.com; 2 School of Nutrition and Health Sciences, College of Public Health and Nutrition, Taipei Medical University, Taipei 110, Taiwan; a19851102@hotmail.com; 3 Department of Pharmacology, School of Medicine, College of Medicine, Taipei Medical University, Taipei 110, Taiwan; hsiehcy@tmu.edu.tw; 4 Department of Nursing, Mackay Junior College of Medicine, Nursing, and Management, Taipei 112, Taiwan; s519@mail.mkc.edu.tw; 5 Division of Thoracic Medicine, Department of Internal Medicine, School of Medicine and School of Respiratory Therapy, College of Medicine, Taipei Medical University, Taipei 110, Taiwan

**Keywords:** endothelin-1, extracellular matrix, mesothelial mesenchymal transition, pleural fibrosis, pleural mesothelial cell, residual pleura thickening, tuberculous pleural effusion

## Abstract

Endothelin (ET)-1 is involved in various fibrotic diseases. However, its implication in pleural fibrosis remains unknown. We aimed to study the profibrotic role of ET-1 in tuberculous pleural effusion (TBPE). The pleural effusion ET-1 levels were measured among 68 patients including transudative pleural effusion (TPE, *n* = 12), parapneumonic pleural effusion (PPE, *n* = 20), and TBPE (*n* = 36) groups. Pleural fibrosis, defined as radiological residual pleural thickening (RPT) and shadowing, was measured at 12-month follow-up. Additionally, the effect of ET-1 on mesothelial mesenchymal transition (MMT) and extracellular matrix (ECM) producion in human pleural mesothelial cells (PMCs) was assessed. Our findings revealed that effusion ET-1 levels were significantly higher in TBPE than in TPE and PPE, and were markedly higher in TBPE patients with RPT >10 mm than those with RPT ≤10 mm. ET-1 levels correlated substantially with residual pleural shadowing and independently predicted RPT >10 mm in TBPE. In PMCs, ET-1 time-dependently induced MMT with upregulation of α-smooth muscle actin and downregulation of E-cadherin, and stimulated ECM production; furthermore, ET receptor antagonists effectively abrogated these effects. In conclusion, ET-1 induces MMT and ECM synthesis in human PMCs and correlates with pleural fibrosis in TBPE. This study confers a novel insight into the pathogenesis and potential therapies for fibrotic pleural diseases.

## 1. Introduction

Endothelin (ET)-1, initially identified as a potent vasoconstrictor, has been substantially implicated in the pathogenesis of inflammation and fibrosis of various organs [1]. In the context of lung diseases, plasma ET-1 levels were elevated in patients with acute respiratory distress syndrome [2], and endothelin blockers reduced lipopolysaccharide-induced lung inflammation in vivo [3]. Additionally, increased expression of ET-1 was observed in lung tissues from patients with idiopathic pulmonary fibrosis (IPF) [4], and ET-1 was reported to recruit and differentiate lung fibroblasts into myofibroblasts, induce alveolar epithelial mesenchymal transition (EMT), and promote extracellular matrix (ECM) production [5,6,7].

Pleural fibrosis restricts lung expansion and results in ventilatory impairment. The etiologies of pleural fibrosis include empyema, tuberculous pleural effusion (TBPE), asbestos pleurisy, improperly drained hemothorax and coronary artery bypass grafting surgery [8]. TBPE remains the most common cause of pleural fibrosis where tuberculosis is endemic. Around 20–50% of patients with TBPE developed clinically significant pleural fibrosis of which the pathogenesis and management remain to be clarified [9,10].

Pleural mesothelial cells (PMCs), a monolayer of cells lining the pleural cavity, actively participate in pleural injury by secreting various cytokines, chemokines and coagulation cascade proteins [11]. Moreover, in response to transforming growth factor (TGF)-β1 and bleomycin, PMCs undergo mesothelial mesenchymal transition (MMT) into myofibroblasts and elaborate excessive ECM, resulting in pleural and subpleural fibrosis [12,13,14,15]. Accordingly, PMCs may play a key contributor in pleural injury and fibrosis.

There is growing evidence of targeting ET-1 as a potential therapy for various inflammatory and fibrotic disorders [16,17]. However, to the best of our knowledge, the role of ET-1 in pleural diseases has never been investigated. The present study aims to assess the profibrotic role of ET-1 in TBPE and to elucidate the effect of ET-1 on MMT in human PMCs in the underlying mechanisms.

## 2. Materials and Methods

### 2.1. Reagents

Heat-killed *Mycobacterium tuberculosis* H37Ra (MTBRa) (Difco Lab, Detroit, MI, USA) and ET-1 (R & D System; Minneapolis, MN, USA) were dissolved in phosphate-buffered saline (PBS) and used as a stimulant [18]. The antibodies to α-smooth muscle actin (α-SMA) and E-cadherin were purchased from Cell Signaling Technology (Beverly, MA, USA), and those to ET-1, collagen I, fibronectin were obtained from R & D System (Minneapolis, MN, USA), Santa Cruz (Dallas, TX, USA), Novus Biologicals (Littleton, CO, USA), respectively. The antibodies to mesothelin, AKT, IκBα and α-tubulin were from Thermo Fisher Scientific (Waltham, MA, USA). The ET receptor antagonists BQ123, BQ788 and Bosentan were acquired from Sigma (St. Louis, MO, USA). Mitogen-activated protein kinase kinase (MEK) inhibitor PD98059, c-Jun N-terminal kinase (JNK) inhibitor SP600125, p38 mitogen-activated protein kinase (MAPK) inhibitor SB203580, nuclear factor (NF)-κB inhibitor parthenolide and phosphatidylinositol 3-kinase (PI3K) inhibitor LY294002 were obtained from Calbiochem (San Diego, CA, USA).

### 2.2. Patient Selection

Consecutive patients with pleural effusion (PE) of unknown cause admitted to Taipei Medical University Hospital were eligible and included if parapneumonic (PPE), tuberculous (TBPE) or transudative PE (TPE) were diagnosed. PPE was defined as a pleural exudate associated with the underlying pneumonia. TBPE was diagnosed by the demonstration of granulomatous pleuritis on closed pleura biopsy specimens with or without the presence of acid-fast bacilli. Ethics approval (CRC-05-11-01) was obtained from the Institutional Review Board of Taipei Medical University Hospital (Taipei, Taiwan). All patients presented written informed consent prior to entering the study. Exclusion criteria included history of invasive pleural procedures, recent severe trauma, hemorrhage or stroke; bleeding diathesis or anticoagulant therapy.

### 2.3. Thoracentesis and Pleural Fluid Analysis

Immediately or within 24 h after hospitalization, 50 mL of pleural fluid was aspirated with the guidance of chest ultrasonography. Pleural fluid analyses, adenosine deaminase (ADA) measurement, microbiological studies were performed routinely. Standard anti-TB medications for 6 months were administered once TBPE was diagnosed.

### 2.4. Chest Radiographs and Pulmonary Function

Posterior-anterior chest radiographs (CXRs) were taken on admission and every 2 months during the follow-up period up to 12 months. The CXR films were read and scored by two radiologists blind to any clinical information to determine (a) lateral pleural thickening: the largest linear width of pleural opacity and (b) CXR score of the size of pleural effusion or thickening: the estimated overall percentage of pleural shadowing in the hemithorax [19]. Clinically significant residual pleural thickening (RPT) was defined as a lateral pleural thickening of >10 mm shown on CXR at the end of 12-month follow-up that was evaluated as pleural fibrosis by chest computed tomography [20]. Pulmonary function tests with spirometry were performed at 12 months following the initiation of treatment.

### 2.5. Measurement of Cytokines and Fibrinolytic Factors in Pleural Effusions

The commercially available enzyme-linked immunosorbent assay kits were used to measure effusion levels of ET-1 (American Diagnostica; Greenwich, CT, USA), tumor necrosis factor (TNF)-α, interleukin (IL)-1β, TGF-β1, plasminogen activator inhibitor (PAI)-1, and tissue plasminogen activator (tPA) (R & D System; Minneapolis, MN, USA) as previously described [19].

### 2.6. Human Pleural Mesothelial Cell (PMC) Culture

The primary human PMCs were harvested from pleural fluids of patients with congestive heart failure and cultured as previously described [19]. The Met-5A human pleural mesothelial cell lines were obtained from American Type Culture Collection (ATCC, Manassas, VA, USA) and grown in medium 199 (GIBCO^TM^, Invitrogen, San Diego, CA, USA) supplemented with 10% fetal bovine serum (FBS) in a humidified atmosphere of 5% CO_2_ at 37 °C as described previously [19].

### 2.7. Western Blot

Proteins were separated by sodium dodecyl sulphate-polyacrylamide gel electrophoresis (SDS-PAGE) and transferred to nitrocellulose membranes. Blotting membranes were incubated with a specific antibody against ET-1, α-SMA, E-cadherin, collagen I and fibronectin, AKT, IκBα and α-tubulin. The quantitative densitometric analysis was conducted as formerly reported [19].

### 2.8. Phase Contrast Microscopy

The cultured primary PMCs were left untreated (control) or treated with ET-1 for various indicated times (24, 48, 72, and 96 h), and the phenotypic changes were determined by phase contrast microscopy [13].

### 2.9. Immunofluorescence Staining

Primary or MeT-5A human PMCs were cultured on collagen (Vitrogen^®^)-coated glass cover slips for 24 h and change to FBS-free medium for another 24 h. Then, for the MMT phenotype study, primary PMCs were treated with or without ET-1 (10 nM) for 72 h and then incubated with primary antibodies: mouse anti-E-cadherin and mouse anti-α-SMA and respective secondary antibody conjugated with fluorescein isothiocyanate (FITC), and viewed by immunofluorescence microscopy.

For evaluation of the effect of ET receptor antagonists on ET-1-induced α-SMA expression, MeT-5A cells were pretreated with PBS, BQ123, BQ788 or Bosentan for 15 min, then treated with ET-1 (10 nM) for 24 h, and incubated with primary antibody to α-SMA. After three washings with PBS, the cells were incubated with secondary FITC-conjugated antibody for 1 h at room temperature.

For measurement of ET-1 expression and MMT in pleural mesothelium, the paraffin-embedded parietal pleura tissue biopsied from patients with TBPE were placed onto glass slides, de-waxed in xylene and alcohols, washed twice with PBS, and then immunostained with primary antibodies to mesothelin, ET-1 or α-SMA, followed by FITC conjugated secondary antibody. Cells were also co-stained with 4’, 6-diamidino-2-phenylindole (DAPI) to visualize nuclei. The stained cells were mounted with anti-fading mounting medium and scanned on the TCS SP5 confocal spectral microscope imaging system (Leica, Wetzlar, Germany) as previously described [21]. 

### 2.10. Reverse Transcription-Polymerase Chain Reaction (RT-PCR)

Total RNA was extracted from cultured PMCs with the TRIsure^®^ reagent (Bioline) and 1 μg RNA was used for cDNA synthesis (SuperScript On-Step reverse transcription-polymerase chain reaction (RT-PCR) system, GIBCOTM). Specific primer sequences (sense/antisense) were designed as follows: α-SMA: 5’-CCAGCTATGTGTGAAGAAGAGG-3’/5’-GTGATCTCCTTCTGCATTCGGT- 3’; GAPDH: 5’-GCCGCCTGGTCACCAGGGCTG-3’/5’-ATGGACTGTGGTCATGAGCCC-3’. PCR products were resolved on agarose gels and bands were identified by ethidium bromide staining.

### 2.11. Statistical Analysis

Quantitative data are presented as median (range) or mean ± SD. Comparisons of continuous data were made using the Kruskal–Wallis test or one way analysis of variance (ANOVA) among three groups, and Mann–Whitney *U* test or unpaired t-test between two groups, where appropriate. The correlations between variables were determined by Spearman rank correlation coefficients. Categorical variables between two groups were examined using the *χ*^2^ method and/or Fisher’s exact test, when appropriate.

Multivariate logistic regression analyses were performed to determine factors independently associated with development of RPT >10 mm. Variables found to be significant in the univariate analysis were entered into a binary logistic regression analysis. Results of multivariable analyses are reported as odds ratios with 95% confidence intervals and p-values. The optimal sensitivity, specificity and cutoff value of pleural fluid variables to predict RPT >10 mm were evaluated with the receiver operating characteristics (ROC) by analyzing the area under the curve. A two-tailed *p*-value < 0.05 was considered to be statistically significant.

## 3. Results

### 3.1. Endothelin (ET)-1 Levels among Transudative Pleural Effusion (TPE), Parapneumonic Pleural Effusion (PPE) and Tuberculous Pleural Effusion (TBPE) Groups

Consecutive 68 patients with TPE (*n* = 12), PPE (*n* = 20) and TBPE (*n* = 36) were enrolled (Table 1), including 42 men and 26 women with an age range from 20 to 95 years. All patients with TPE were diagnosed with congestive heart failure, and the PPE group consisted of 8 uncomplicated PPE and 12 complicated PPE. All patients finished 12 months of follow-up from January 2011 through December 2014.

**Figure 1:**
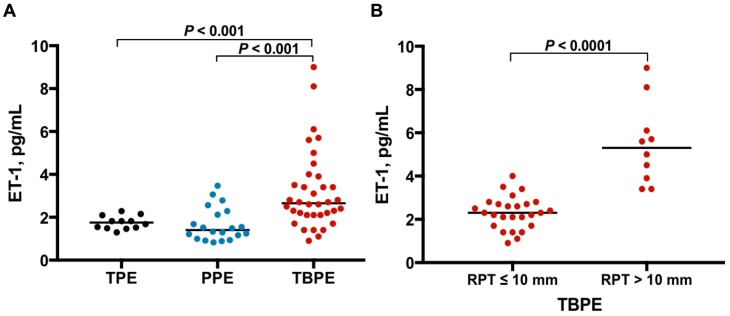
ET-1 levels among TPE, PPE and TBPE. (**A**) Pleural effusion ET-1 levels were significantly higher in TBPE than in TPE and PPE. (**B**) Pleural fluid ET-1 levels were significantly higher in TBPE patients with RPT >10 mm (*n* = 10) than in those with RPT ≤10 mm (*n* = 26). ET-1, endothelin-1; TPE, transudative pleural effusion (*n* = 12); PPE, parapneumonic pleural effusion (*n* = 20); TBPE, tuberculous pleural effusion (*n* = 36); RPT, residual pleural thickening.

As shown in Table 1 and Figure 1A, the median levels of effusion ET-1 were significantly higher in TBPE group (2.7 pg/mL, range 0.9–9.0 pg/mL) than in TPE (1.8 pg/mL, range 1.3–2.3 pg/mL) and PPE (1.4 pg/mL, range 0.8–3.5 pg/mL) groups (TBPE vs. TPE, *p* < 0.001; TBPE vs. PPE, *p* < 0.001), while the ET-1 values were comparable between TPE and PPE groups. This result suggests that ET-1 plays a distinctive role in TBPE rather than in TPE or PPE.

### 3.2. ET-1 Levels between TBPE Patients with Residual Pleural Thickening (RPT) ≤10 mm and RPT >10 mm

Subsequently, to investigate the profibrotic role of ET-1 in TBPE, the TBPE patients were classified into RPT ≤10 mm (*n* = 26) and RPT >10 mm (*n* = 10) groups, based on the CXR at the end of 12-month follow-up. As shown in Figure 1B, the effusion ET-1 level was remarkably higher in TBPE patients with RPT >10 mm (4.9 pg/mL, range 3.4–9.0 pg/mL) than those with RPT ≤10 mm (2.3 pg/mL, range 0.9–4.0 pg/mL) (*p* < 0.0001).

### 3.3. Cytokines and Fibrinolytic Factors between TBPE Patients with RPT ≤10 mm and RPT >10 mm

In pleural fluid analysis, the levels of pH, glucose and LDH reflect the degree of inflammation [22], the balance of PAI-1 and tPA determines the fibrinolytic activity [11], the proinflammatory cytokines TNF-α and IL-1β accentuate inflammatory responses [23], and the profibrotic cytokine TGF-β1 mediates fibrosis in the pleural space [12]. The effusion ADA level is useful for diagnosis of TBPE [10], although its relationship with pleural fibrosis remains unknown. Therefore, we further compared these pleural fluid characteristics, ADA, TNF-α, IL-1β, TGF-β1 and fibrinolytic factors PAI-1 and tPA between the two groups (Table 2). The pleural fluid parameters demonstrated that RPT >10 mm group had significantly lower levels of effusion pH and ADA than did RPT ≤10 mm group, while there was no considerable difference in pleural fluid values of glucose, LDH, and leukocyte count between two groups. Moreover, besides ET-1, the effusion levels of PAI-1, TNF-α, IL-1β, and especially TGF-β1 were significantly higher in RPT >10 mm group than in RPT ≤10 mm group. Additionally, the former had greater initial effusion CXR score on presentation than the latter. Furthermore, patients with RPT >10 mm had significant lower forced vital capacity than did those with RPT ≤10 mm at 12 months. These findings imply that the increased inflammation, decreased fibrinolysis and elevated profibrotic cytokines, including ET-1 and TGF-β1, may be associated with the development of pleural fibrosis in TBPE.

### 3.4. Correlation between ET-1 and Inflammatory Parameters, Fibrinolytic Factors and Cytokines in TBPE

Accordingly, to explore the link between ET-1 and inflammation, fibrinolysis and fibrosis in TBPE, we examined the relationship between ET-1 and inflammatory parameters, fibrinolytic factors and other cytokines among TBPE patients (Table 3). The results demonstrated that the effusion levels of ET-1 were positively correlated with those of TGF-β1 (*r* = 0.45, *p* = 0.008), and negatively correlated with those of glucose (*r* = −0.35, *p* = 0.036). However, there was no significant correlation between ET-1 and pH, LDH, PAI-1, tPA, TNF-α and IL-1β, respectively. The current data suggest that ET-1 may have greater association with fibrosis than with inflammation or fibrinolysis in TBPE.

### 3.5. Multivariate Logistic Regression Analysis

To further ascertain the importance of ET-1 in fibrosis of TBPE, multivariate logistic regression analysis was used to identify the factors associated with RPT >10 mm in TBPE after 12-month follow-up (Table 4). Variables of significance in univariate analysis were included for analysis and ET-1 and TGF-β1 were treated separately because they were mutually correlated (Table 3). This demonstrated that only higher effusion ET-1 and TGF-β1 levels were independent predictors for RPT >10 mm in TBPE.

### 3.6. Optimal Sensitivity, Specificity and Cutoff Value of Variables to Predict RPT >10 mm

**Figure 2:**
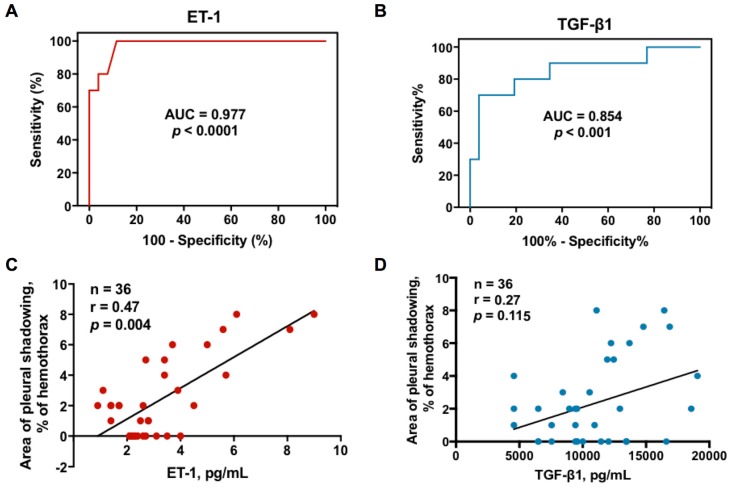
Relationship between effusion ET-1 or TGF-β1 and residual pleural fibrosis in TBPE: (**A**) receiver operating characteristic (ROC) curve for effusion ET-1 level to predict RPT >10 mm in TBPE; (**B**) ROC curve for effusion TGF-β1 level to predict RPT >10 mm in TBPE; (**C**) Correlation between effusion ET-1 level and residual pleural shadowing CXR score in patients with TBPE; (**D**) Correlation between effusion TGF-β1 level and residual pleural shadowing CXR score in patients with TBPE. AUC, area under the ROC curve; ET-1, endothelin-1; TGF-β1, transforming growth factor-β1; RPT, residual pleural thickening; TBPE, tuberculous pleural effusion.

Furthermore, the ROC curves showed that the effusion ET-1 at the cutoff level >3.7 pg/mL had highest sensitivity and specificity for predicting RPT >10 mm in TBPE patients (area under the ROC curve = 0.977, 95% confidence interval (CI) = 0.938–1.016; sensitivity 80%, 95% CI = 44.3–97.5%; specificity 96.2%, 95% CI = 80.4–99.9%) (Figure 2A), followed by TGF-β1 at the cutoff level >13,452.7 pg/mL (area under the ROC curve = 0.854, 95% CI = 0.697–1.011; sensitivity 70%, 95% CI = 34.8–93.3%; specificity 96.2%, 95% CI = 80.4–99.9%) (Figure 2B).

### 3.7. Correlation between Effusion ET-1, TGF-β1 and Residual Pleural Fibrosis

Given the predictive value of effusion ET-1 and TGF-β1, the correlation between ET-1, TGF-β1 and residual pleural fibrosis were analyzed. As shown in Figure 2C,D, the effusion levels of ET-1 were positively correlated with the CXR score of pleural shadowing at 12 months; however, there was no significant correlation between TGF-β1 and residual pleural shadowing, suggesting that ET-1 is substantially implicated in fibrogenesis of TBPE that merits further experiments to confirm.

### 3.8. Heat-Killed Mycobacterium tuberculosis H37Ra (MTBRa) Induces ET-1 Production in Human PMCs

**Figure 3:**
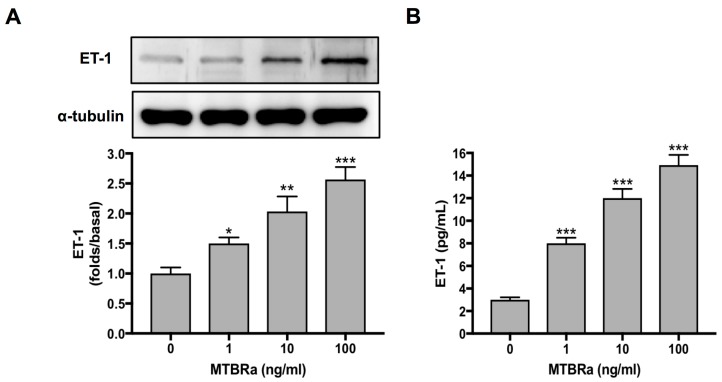
MTBRa induces ET-1 production in primary human PMCs. Cultured primary human PMCs (5 × 10 ^5^ cells) were stimulated with the indicated concentrations of MTBRa for 24 h. (**A**) The total proteins from cell lysates were analyzed through Western blotting. (**B**) The levels of ET-1 in the culture supernatants were analyzed by ELISA. Data were shown as mean ± SD of three independent experiments. * *p* < 0.05, ** *p* < 0.01 and *** *p* < 0.001 compared with resting group. MTBRa, heat-killed *Mycobacterium tuberculosis* H37Ra; PMC, pleural mesothelial cell.

To elucidate the marked increase of ET-1 in TBPE (Figure 1A), we studied the ET-1 production by primary human PMCs upon MTBRa stimulation. As shown in Figure 3A, ET-1 expression increased significantly upon stimulation with various concentrations of MTBRa (0.1, 1, 10 and 100 ng/mL) for 24 h. Consistently, the ET-1 level in the supernatants of PMC culture stimulated with MTBRa for 24 h increased in a concentration-dependent manner as compared with the control (Figure 3B). These findings suggest that, upon *Mycobacterium tuberculosis* (*M. tuberculosis*) infection, human PMCs are activated to produce a considerable amount of ET-1 that contribute to the elevated levels in TBPE.

### 3.9. ET-1 Induces MMT and ECM Synthesis in PMCs

**Figure 4:**
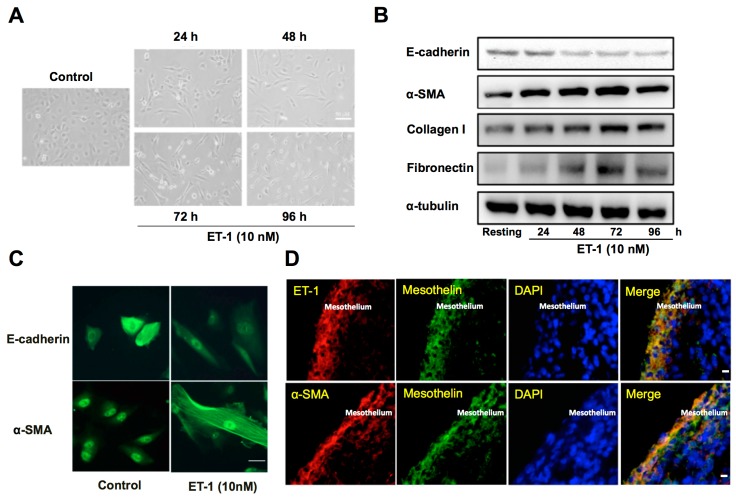
ET-1 induces MMT and ECM synthesis in primary human PMCs and associates with α-SMA expression in pleural mesothelium of patients with TBPE. (**A**) Primary human PMCs were treated with or without (control) ET-1 for 96 h. Phenotypic change (transition from mesothelial toward mesenchymal phenotype) was evaluated by phase contrast microscopy. Scale bar, 50 μm. (**B**) Primary human PMCs were treated with ET-1 (10 nM) for the indicated times, and E-cadherin, α-SMA, collagen I and fibronectin were analyzed by Western blotting. Results are representative of three independent experiments. (**C**) Primary human PMCs were treated with or without (control) ET-1 for 72 h, and phenotypic change and expression for E-cadherin and α-SMA were evaluated by immunofluorescence microscopy. Scale bar, 20 μm. (**D**) Immunofluorescence staining for ET-1 (red) or α-SMA (red) and mesothelin (green) in PMCs was performed on paraffin-embedded pleural biopsy specimens from patients with TBPE and observed by confocal microscopy. Cells were also co-stained with DAPI to visualize the nuclei (blue). Scale bar, 50 μm. MMT, mesothelial mesenchymal transition; ECM, extracellular matrix; PMC, pleural mesothelial cell; α-SMA, α-smooth muscle actin; DAPI, 4’, 6-diamidino-2- phenylindole.

Subsequently, to verify the profibrotic effect of ET-1 on pleural mesothelium, the human PMCs were treated with or without 10 nM of ET-1 for 96 h. The primary human PMCs in the absence of ET-1 (control) showed typical cobblestone appearance, whereas ET-1-treated PMCs revealed substantial phenotypic change after 24 h and transformed into fibroblast-like appearance with elongated, spindle-shaped morphology (Figure 4A). Consistently, ET-1 significantly increased α-SMA while decreased E-cadherin expression in PMCs, compared to the resting condition (Figure 4B). Additionally, to further validate that the MMT will lead to ECM synthesis, we also examined the expression of collagen I and fibronectin in PMCs activated with ET-1. A significant increase in the production of collagen I and fibronectin was noted after 24 h treatment of ET-1 in a time-dependent manner, with maximal response occurring at 72 h (Figure 4B). Similarly, the immunofluorescence staining for E-cadherin and α-SMA revealed MMT phenotypic change and downregulation of E-cadherin and upregulation of α-SMA in ET-1-treated PMCs compared with the resting controls (Figure 4C). These findings indicated that ET-1 stimulates human PMCs to undergo MMT and produce ECM and thereby contributes to pleural fibrosis.

### 3.10. Expression of ET-1 and α-SMA in the Pleural Mesothelium of TBPE

Furthermore, the immunofluorescence staining was performed on paraffin-embedded pleural biopsy specimens from patients with TBPE (Figure 4D), and demonstrated proliferation of PMCs (mesothelin positive cells) into multilayers that exhibited substantial dual expression of ET-1^+^/mesothelin^+^ and α-SMA^+^/mesothelin^+^, which further indicates the important implication of ET-1 and MMT in the pathogenesis of TBPE.

### 3.11. MTBRa-Induced Mesothelial Mesenchymal Transition (MMT) and Extracellular Matrix (ECM) Synthesis in PMCs via ET-1

**Figure 5:**
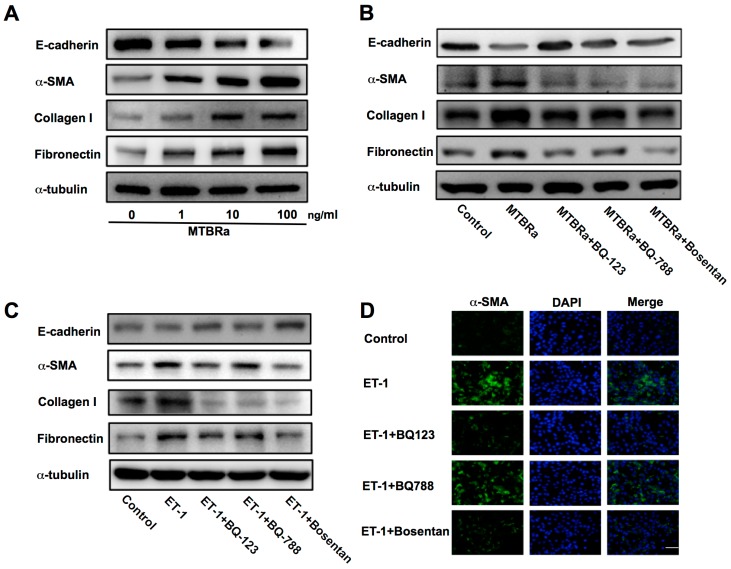
ET-1 mediates MTBRa-induced MMT in human PMCs mainly through ET-A receptor. (**A**) MeT-5A cells were treated with or without (control) indicated concentrations of MTBRa for 24 h, and E-cadherin, α-SMA, collagen I and fibronectin were analyzed by Western blotting. (**B**) MeT-5A cells were pretreated with PBS, BQ123 (10 μM), BQ788 (10 μM) or Bosentan (10 μM) for 15 min, then treated with or without (control) MTBRa (10 ng/mL) for 24 h, and E-cadherin, α-SMA, collagen I and fibronectin were analyzed by Western blotting. (**C**) MeT-5A cells were pretreated with PBS, BQ123 (10 μM), BQ788 (10 μM) or Bosentan (10 μM) for 15 min, then treated with or without (control) ET-1 (10 nM) for 24 h, and E-cadherin, α-SMA, collagen I and fibronectin were analyzed by Western blotting. (**D**) MeT-5A cells were pretreated with PBS, BQ123 (10 μM), BQ788 (10 μM) or Bosentan (10 μM) for 15 min, then treated with or without (control) ET-1 (10 nM) for 24 h, and immunofluorescence staining for α-SMA (green) was evaluated by confocal microscopy. Cells were also co-stained with DAPI to visualize the nuclei (blue). Scale bar, 20 μm. Data are representative of three independent experiments. MTBRa, heat-killed *Mycobacterium tuberculosis* H37Ra MMT, mesothelial mesenchymal transition; ECM, extracellular matrix; α-SMA, α-smooth muscle actin; DAPI, 4’, 6-diamidino-2- phenylindole.

Accordingly, MTBRa was used to stimulate PMCs to investigate the role of ET-1 and MMT in TBPE. It demonstrated that MTBRa concentration-dependently reduced E-cadherin expression, induced α-SMA synthesis, and elicited production of collagen I and fibronectin in MeT-5A cells (Figure 5A). Conversely, pretreatment with the ET-A receptor antagonist (BQ-123), ET-B receptor antagonist (BQ-788) and dual ET receptor antagonist (Bosentan) all abrogated the decrease in E-cadherin and increase in α-SMA, collagen I and fibronectin caused by MTBRa (Figure 5B). Together, these findings indicate that MTBRa stimulates MMT and ECM production in PMCs via the action of ET-1.

### 3.12. ET-1 Induces MMT in PMCs Mainly through Activation of ET-A Receptor

Furthermore, we explored which ET receptor subtype mediates the action of ET-1 on MMT in PMCs. The data showed that pretreatment with BQ-123 or Bosentan significantly attenuated ET-1−induced downregulation of E-cadherin and upregulation of α-SMA, while BQ-788 had the same effect but to a lesser degree (Figure 5C). In parallel, the immunofluorescence study revealed that both BQ-123 and Bosentan, while BQ-788 less prominently, inhibited the ET-1−stimulated increase in α-SMA expression (Figure 5D). Moreover, treatment with three ET receptor antagonists all substantially ablated the expression of ECM proteins (Figure 5C). These results indicate that ET-1 induces MMT predominantly through activation of ET-A receptor.

### 3.13. ET-1−Induced α-SMA Expression is PI3K/AKT and NF-κB Dependent

**Figure 6:**
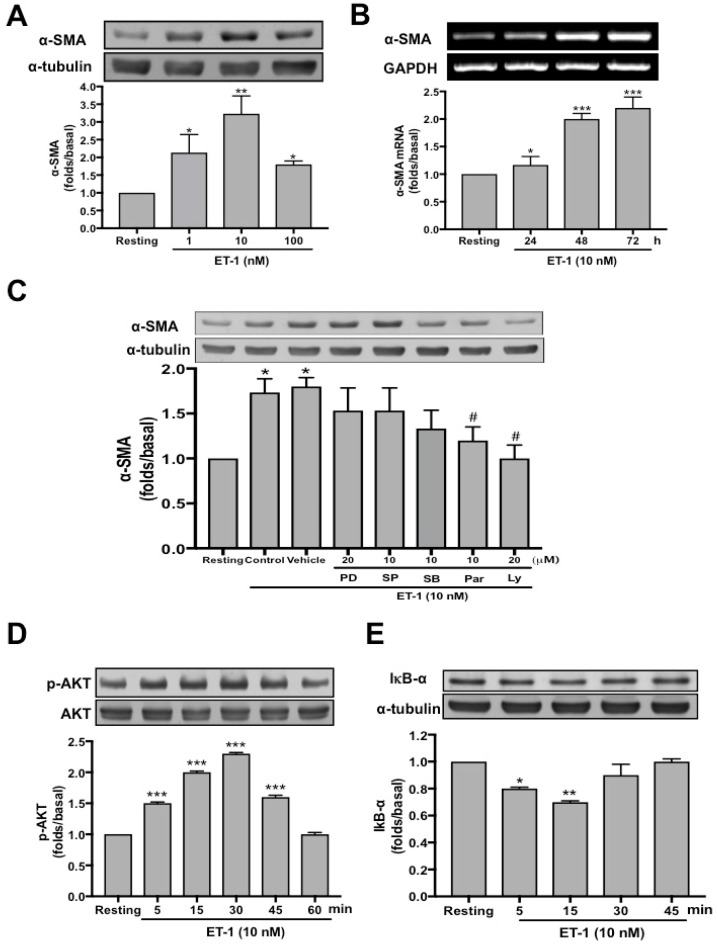
ET-1−induced α-SMA expression in human PMCs is PI3K/AKT and NF-κB dependent. (**A**) Primary human PMCs were treated with or without (resting) the indicated concentration of ET-1 for 72 h, and α-SMA was analyzed by Western blotting. (**B**). Primary human PMCs were treated with ET-1 10 nM for the indicated times, and the mRNA expressions of α-SMA were analyzed by semiquantitative reverse transcriptase PCR. (**C**) MeT-5A cells were preincubated with the vehicle, PD98059 (PD, 20 μM), SP600125 (SP, 10 μM), SB203580 (SB, 10 μM), Parthenolide (Par, 10 μM) or LY294002 (LY, 20 μM), then ET-1 was added for 24 h, and α-SMA was analyzed by Western blotting. (**D**,E****) MeT-5A cells were treated without (resting) or with ET-1 10 nM for the indicated times, and phosphorylated AKT, total AKT or IκB-α were analyzed by Western blotting. The relative folds of densitometric data were expressed as the mean ± SD of three independent experiments. * *p* < 0.05, ** *p* < 0.01 and *** *p* < 0.001 compared with resting group; ^#^ *p* < 0.05 compared with vehicle group. ET-1, endothelin-1; AKT, protein kinase B; α-SMA, α-smooth muscle actin; PI3K, phosphatidylinositol 3-kinases; GAPDH, glyceraldehyde 3-phosphate dehydrogenase; IκB-α, inhibitor of κB-α.

**Figure 7:**
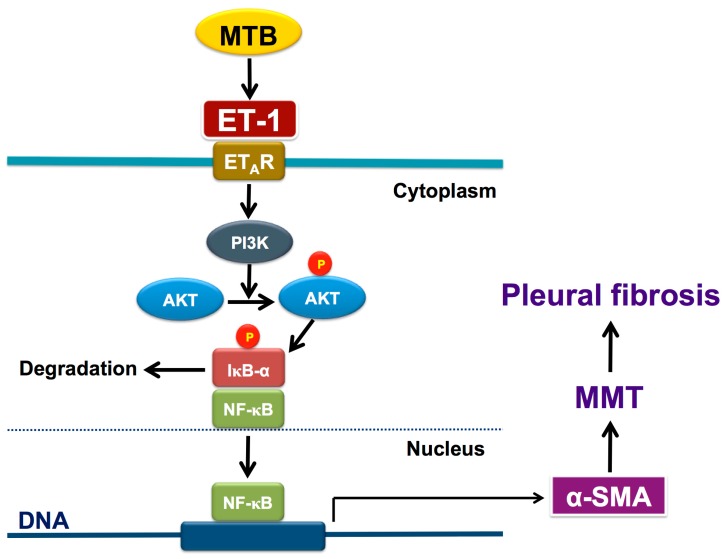
Schematic diagram of the activated signalings of MMT in human pleural mesothelial cells upon *Mycobacterium tuberculosis* infection and ET-1 stimulation. *Mycobacterium tuberculosis* induces production of ET-1 that binds to ET-A receptor and results in phosphorylation of PI3K/AKT and activation of NF-κB, leading to expression of α-SMA and subsequent MMT and pleural fibrosis (see text for further explanation). ^℗^ phosphorylated; AKT, protein kinase B; α-SMA, α-smooth muscle actin; ET-1, endothelin-1; ET_A_R, ET-A receptor; IκB-α, inhibitor of κB-α; MMT, mesothelial mesenchymal transition; MTB, *Mycobacterium tuberculosis*; NF-κB, nuclear factor-κB; PI3K, phosphatidylinositol 3-kinases.

To further investigate the mechanism underlying the ET-1−activated MMT in PMCs, we proceeded to identify the involved cellular signaling. The data demonstrated ET-1 significantly induced α-SMA in MeT-5A cells in a concentration-dependent manner with a maximal response occurring with 10 nM (Figure 6A). Besides, to recognize whether the increase in α-SMA protein expression after ET-1 stimulation resulted from the induction of α-SMA mRNA expression, RT-PCR analysis was performed. After treatment with ET-1 for 24, 48, and 72 hours, respectively, MeT-5A cells had significant increase in α-SMA mRNA expression at the concentration of 10 nM ET-1 (Figure 6B). Furthermore, as shown in Figure 6C, pretreatment with neither an MEK inhibitor (PD98059; 20 μM), a JNK inhibitor (SP600125; 10 μM) nor a p38 MAPK inhibitor (SB203580; 10 μM) attenuated α-SMA expression induced by ET-1. In contrast, a NF-κB inhibitor (Parthenolide; 10 μM) and a PI3K inhibitor (LY294002; 20 μM) significantly reduced ET-1−stimulated α-SMA protein production. Consistently, ET-1 significantly induced AKT phosphorylation (Figure 6D) and inhibitor of κB-α (IκB-α) degradation (Figure 6E) in a time-dependent manner. Collectively, these findings demonstrated that ET-1 induced MMT in PMCs is PI3K/AKT and NF-κB dependent and possibly through activation of ET-A receptor and PI3K/AKT/NF-κB signaling (Figure 7).

## 4. Discussion

The present study demonstrated that the levels of effusion ET-1 were significantly higher in TBPE than in TPE and PPE, and markedly greater in TBPE patients with RPT >10 mm than those with RPT ≤10 mm. Moreover, ET-1 levels correlated significantly with residual pleural shadowing of TBPE and could predict pleural fibrosis at the end of 12-month follow-up. The in vitro experiments showed that MTBRa significantly stimulated human PMCs to produce ET-1 which time-dependently induced MMT with downregulation of E-cadherin and upregulation of α-SMA, collagen I and fibronectin in human PMCs, whereas pretreatment with ET-A receptor antagonist substantially attenuated all the effects. Moreover, concomitant expression of ET-1 and α-SMA was demonstrated in the pleural mesothelium of patients with TBPE, and ET-1 significantly increased α-SMA expression through triggering AKT phosphorylation and NF-κB activation. To our knowledge, this is the first study to signify the expression and profibrotic implication of ET-1 and MMT in TBPE and to demonstrate the regulatory mechanism underlying the ET-1−induced MMT and ECM production in human PMCs.

ET-1 has been associated with the pathogenesis of numerous infectious diseases [24], though its role in pleural infections remains elusive. Besides, the link between ET-1 and *M. tuberculosis* infection has rarely been explored. A previous in vivo study showed that the blockade of ET-1 activity by antagonism might lead to progression of tuberculous lung lesions, indicating that ET pathways play a regulatory role in *M. tuberculosis* infection [25]. We first demonstrated that ET-1 level was markedly higher in TBPE than in TPE, while not significantly elevated in PPE, and that MTBRa induced substantial production of ET-1 in human PMCs, suggesting the distinctive pathogenic role of ET-1 in TBPE.

To ascertain the involvement of ET-1 in the inflammation, fibrinolysis and fibrosis in TBPE, correlation analysis was conducted and showed that ET-1 correlated positively with the most potent profibrotic cytokine TGF-β1. However, no significant correlation existed between ET-1 and inflammatory parameters, fibrinolytic factors and proinflammatory cytokines, respectively. Accordingly, to further clarify the implication of ET-1 in pleural fibrosis, we measured the residual pleural opacity of all TBPE patients and found that both effusion ET-1 and TGF-β1 were remarkably higher in patients with RPT >10 mm than those with RPT ≤10 mm. Moreover, ET-1, compared to TGF-β1, was a better predictor for RPT >10 mm and had stronger positive correlation with the area of residual pleural opacity of TBPE. Nonetheless, as studies have demonstrated the mutual regulation and collaboration between TGF-β1 and ET-1 in the development of pulmonary fibrosis [26], the current data does not imply the superiority of ET-1 over TGF-β1 in the modulation of pleural fibrogenesis, but features the importance of ET-1 in TBPE. Taken together, these findings suggest that ET-1, although not linked to increased inflammation or disordered fibrinolysis, is essential for tuberculous pleural fibrosis, probably acting via induction of aberrant cellular response to injury such as cellular mesenchymal transition and ECM overproduction [5,6,7].

Increasing evidence demonstrated that PMCs experience mesenchymal transition upon stimulation by TGF-β1 or bleomycin [12,13,14,15]. In parallel, we demonstrated that ET-1 time−dependently induced protein expression of the myofibroblast−specific marker, α-SMA, while reduced the epithelial cell marker, E-cadherin, and led to phenotypic alteration in primary human PMCs. Concomitantly, ET-1 stimulated human PMCs to produce collagen I and fibronectin that further verify the fibrogenic activity of PMCs in response to ET-1. Moreover, we demonstrated the concurrent expression of ET-1 and α-SMA in the pleural mesothelium of patients with TBPE and that MTBRa stimulated MMT and ECM synthesis in PMCs through the actions of ET-1. Collectively, these results indicate that ET-1 is an important mediator for MMT in PMCs and may, thereby, contribute to pleural fibrosis in TBPE.

We further investigated the involved receptors and signalings mediating the ET-1−induced MMT and showed that treatment with ET-A receptor antagonist (BQ-123) effectively reversed the loss of E-cadherin and gain of α-SMA in PMCs stimulated by ET-1, while ET-B receptor antagonist (BQ-788) had a similar but lesser effect. This may be due to either lower affinity for or diminished amount of the ET-B receptor on PMCs. Nonetheless, both ET-A and ET-B blockers efficiently repressed the production of collagen I and fibronectin, which may suggest the distinct regulation between MMT and ECM synthesis in PMCs upon ET-1 activation. Furthermore, ET-1 activated PI3K/AKT and NF-κB signaling and blockade of PI3K/AKT and NF-κB significantly attenuated ET-1–upregulated α-SMA expression in PMCs. Altogether, these findings, in line with previous reports [5,6,15], indicated that ET-1 induces MMT in PMC mainly through activation of ET-A receptor and is PI3K/AKT and NF-κB dependent.

As for the therapeutic implication of ET-1 for pleural fibrosis, previous reports have demonstrated that ET receptor antagonist treatment efficiently attenuated ET-1–mediated alveolar EMT in vitro [5,6], mitigated bleomycin-induced lung fibrosis in vivo [27], and displayed a favorable trend to improve progression free survival of histology–proven IPF patients [28,29]. Additionally, recent in vivo studies have employed the heme oxygenase-1 inducer, carbon monoxide or glycogen synthase kinase-3β inhibitor, to attenuate MMT in PMCs that significantly abrogated pleural fibrosis [30,31]. In parallel, our data signified the antifibrotic potential of ET receptor antagonism by concurrently reversing MMT and reducing ECM production in human PMCs. However, the pleural biopsy specimens from patients at diagnosis of TBPE revealed that the MMT of pleural mesothelium occurred early in the disease process (Figure 4D), implying that prevention of MMT may not be feasible. Nevertheless, the reversing effect of ET blockers on MMT (Figure 5B–D) may infer a therapeutic potential. Accordingly, more in vivo and clinical studies to inhibit ET-1 as well as MMT are needed for developing novel agents for treatment of pleural fibrosis. Collectively, the current study highlights the role of ET-1 and MMT in the fibrogenesis of TBPE and warrants further investigations of targeting ET-1 and/or MMT as treatment strategies for pleural fibrosis.

## 5. Conclusions

In conclusion, ET-1 induces MMT and ECM synthesis in human PMCs and correlates with pleural fibrosis in TBPE. This study confers a novel insight into the pathogenesis and potential therapies for fibrotic pleural diseases.

## Figures and Tables

**Table 1:** Demographics and pleural fluid characteristics and ET-1 levels among all patients (*n* = 68) ^†^.

	TPE	PPE	TBPE
Subject, *n*	12	20	36
Age, years	83 (54–95)	64 (32–86)	78 (20–93)
Males, *n*	7	13	22
Symptom onset to enrollment, days	7 (6–11)	7 (6–10)	7 (5–9)
Pleural fluid			
pH value	7.38 (7.32–7.42)	7.25 (6.45–7.50)	7.36 (6.90–7.51)
Glucose, mg/dL	110 (73–152)	98 (5–288)	122 (16–188)
Protein, g/L	2.9 (1.3–4.2)	4.3 (3.4–6.0)	4.8 (1.8–6.4)
LDH, IU/dL	109 (57–135)	339 (62–7152)	293 (64–1999)
Leukocyte count, cells/μL	382 (115–490)	2805 (72–42250)	1044 (81–15840)
ADA, IU/L	26 (6–39)	62 (21–130)	124 (48–262)
ET-1, pg/mL	1.8 (1.3–2.3)	1.4 (0.8–3.5)	2.7 (0.9–9.0)
mean (SD)	1.8 (0.3)	1.7 (0.8)	3.2 (1.8)

Definition of abbreviations: ET-1, endothelin-1; TPE, transudative pleural effusion; PPE, parapneumonic effusion; TBPE, tuberculous pleural effusion; LDH, lactate dehydrogenase; ADA, adenosine deaminase. ^†^ Data expressed as median (range) unless specified.

**Table 2:** Demographics, pleural fluid characteristics, and effusion levels of fibrinolytic factors and cytokines between TBPE patients with RPT ≤10 mm and RPT >10 mm ^†^.

	RPT ≤10 mm	RPT >10 mm	*p*-Value
Subject, *n*	26	10	
Age, years	76 (20–93)	83 (34–91)	0.162
Males, *n*	20	8	0.999
Symptom onset to enrollment, days	10 (7–21)	11 (8–30)	0.183
Pleural fluid			
pH value	7.40 (7.00–7.50)	7.30 (6.90–7.40)	0.014
Glucose, mg/dL	134 (51–188)	90 (16–179)	0.070
Protein, g/dL	4.3 (1.8–5.8)	4.6 (2.4–6.4)	0.230
LDH, IU/dL	274 (93–1280)	442 (64–1999)	0.124
Leukocyte count, cells/μL	1044 (81–15849)	933 (114–6760)	0.538
ADA, IU/L	125 (48–262)	80 (59–157)	0.036
ET-1, pg/mL	2.3 (0.9–4.0)	4.9 (3.4–9.0)	<0.0001
PAI-1, ng/mL	101.9 (24.4–268.0)	114.5 (101.9–234.0)	0.012
tPA, ng/mL	23.3(1.4–62.7.0)	16.0 (1.0–31.6)	0.132
TNF-α, pg/mL	43.2 (12.9–223.6)	87.7 (34.9–250.0)	0.034
IL-1β, pg/mL	4.5 (2.3–33.2)	15.5 (2.8–53.1)	0.043
TGF-β1, pg/mL	9546.7 (4567.0–16605.3)	14243.8 (8433.4–19060.0)	<0.001
Initial pleural effusion CXR score, %	41 (19–64)	66 (46–98)	<0.001
Residual pleural shadowing CXR score, %	0.5 (0–5.0)	6.0 (2.0–8.0)	<0.0001
FVC at 12 months, % predicted	81 (79–82)	73 (70–76)	<0.001

Definition of abbreviations: TBPE, tuberculous pleural effusion; RPT, residual pleural thickening; LDH, lactate dehydrogenase; ADA, adenosine deaminase; ET-1, endothelin-1; PAI-1, plasminogen activator inhibitor-1; tPA, tissue-type plasminogen activator; TNF-α, tumor necrosis factor-α; IL-1β, interleukin-1β; TGF-β1, transforming growth factor-β1; CXR, chest radiograph; FVC, forced vital capacity. ^†^ Data expressed as median (range).

**Table 3:** Correlation between ET-1 and inflammatory parameters, fibrinolytic factors and cytokines in TBPE (*n* = 36).

Variables	Coefficient *	*p*-Value
pH value	−0.11	0.532
Glucose, mg/dL	−0.35	0.036
LDH, IU/dL	−0.12	0.468
PAI-1, ng/mL	0.17	0.327
tPA, ng/mL	−0.22	0.207
TNF-α, pg/mL	0.19	0.277
IL-1β, pg/mL	0.01	0.959
TGF-β1, pg/mL	0.45	0.008

Definition of abbreviations: ET-1, endothelin-1; TBPE, tuberculous pleural effusion; LDH, lactate dehydrogenase; PAI-1, plasminogen activator inhibitor-1; tPA, tissue-type plasminogen activator; TNF-α, tumor necrosis factor-α; IL-1β, interleukin-1β; TGF-β1, transforming growth factor-β1. * Spearman correlation coefficient.

**Table 4:** Multivariate logistic regression analyses of factors associated with RPT >10 mm among TBPE patients (*n* = 36).

Variables	ET-1 Excluded			TGF-β1 Excluded		
	OR	95% CI	*p*-Value	OR	95% CI	*p*-Value
ET-1, pg/mL				52.78	1.30–2147.95	0.006
pH value	0.91	0.89–1.00	0.116	0.02	0.01–1.21	0.716
ADA, IU/L	1.26	0.76–1.51	0.609	1.01	0.99–1.02	0.961
PAI-1, ng/mL	1.05	0.98–1.23	0.310	1.02	0.98–1.03	0.135
TNF-α, pg/mL	1.01	0.98–1.04	0.215	1.01	0.99–1.02	0.059
IL-1β, pg/mL	1.00	0.99–1.00	0.123	1.06	0.99–1.12	0.104
TGF-β1, pg/mL	1.12	1.03–1.23	0.018			

Definition of abbreviations: RPT, residual pleural thickening; TBPE, tuberculous pleural effusion; OR; odds ratio; CI, confidence interval; ET-1, endothelin-1; ADA, adenosine deaminase; PAI-1, plasminogen activator inhibitor-1; TNF-α, tumor necrosis factor-α; IL-1β, interleukin-1β; TGF-β1, transforming growth factor-β1.
